# A Systematic Analysis of the Peripheral and CNS Effects of Systemic LPS, IL-1Β, TNF-α and IL-6 Challenges in C57BL/6 Mice

**DOI:** 10.1371/journal.pone.0069123

**Published:** 2013-07-01

**Authors:** Donal T. Skelly, Edel Hennessy, Marc-Andre Dansereau, Colm Cunningham

**Affiliations:** School of Biochemistry and Immunology and Trinity College Institute of Neuroscience, Trinity College, Dublin, Republic of Ireland; Virginia Commonwealth University, United States of America

## Abstract

It is increasingly clear that systemic inflammation has both adaptive and deleterious effects on the brain. However, detailed comparisons of brain effects of systemic challenges with different pro-inflammatory cytokines are lacking. In the present study, we challenged female C57BL/6 mice intraperitoneally with LPS (100 µg/kg), IL-1β (15 or 50 µg/kg), TNF-α (50 or 250 µg/kg) or IL-6 (50 or 125 µg/kg). We investigated effects on core body temperature, open field activity and plasma levels of inflammatory markers at 2 hours post injection. We also examined levels of hepatic, hypothalamic and hippocampal inflammatory cytokine transcripts. Hypothermia and locomotor hypoactivity were induced by LPS>IL-1β>TNF-α>>IL-6. Systemic LPS, IL-1β and TNF-α challenges induced robust and broadly similar systemic and central inflammation compared to IL-6, which showed limited effects, but did induce a hepatic acute phase response. Important exceptions included IFNβ, which could only be induced by LPS. Systemic IL-1β could not induce significant blood TNF-α, but induced CNS TNF-α mRNA, while systemic TNF-α could induce IL-1β in blood and brain. Differences between IL-1β and TNF-α-induced hippocampal profiles, specifically for IL-6 and CXCL1 prompted a temporal analysis of systemic and central responses at 1, 2, 4, 8 and 24 hours, which revealed that IL-1β and TNF-α both induced the chemokines CXCL1 and CCL2 but only IL-1β induced the pentraxin PTX3. Expression of COX-2, CXCL1 and CCL2, with nuclear localisation of the p65 subunit of NFκB, in the cerebrovasculature was demonstrated by immunohistochemistry. Furthermore, we used cFOS immunohistochemistry to show that LPS, IL-1β and to a lesser degree, TNF-α activated the central nucleus of the amygdala. Given the increasing attention in the clinical literautre on correlating specific systemic inflammatory mediators with neurological or neuropsychiatric conditions and complications, these data will provide a useful resource on the likely CNS inflammatory profiles resulting from systemic elevation of particular cytokines.

## Introduction

The dogma of an ‘immune-privileged’ CNS has been supplanted over the past twenty years as a result of many studies that demonstrate intricate interactions between the immune and nervous systems [1]. It is now clear that systemic inflammation induced by conserved pathogen-associated molecular patterns (PAMPs) such as lipopolysaccharide (LPS) and double stranded RNA, along with pro-inflammatory mediators, can trigger CNS inflammation [2]. PAMPs, prostaglandins and induced pro-inflammatory cytokines transduce systemic inflammation into the CNS via parallel neural and humoral routes. The afferent vagal and trigeminal nerves, activated by locally synthesised cytokines, can mediate brainstem, hypothalamic and limbic activation. Cells along the vasculature of the brain, such as endothelial cells and perivascular macrophages, can be activated by circulating PAMPs and macrophage-generated pro-inflammatory mediators. Circulating inflammatory molecules can also be transported by soluble receptors or diffuse directly into the CNS at circumventricular organs [3].

Central inflammatory mediators resulting from systemic inflammation, acting primarily on the hypothalamus, are known to produce a coordinated set of adaptive changes, collectively known as sickness behaviour. This motivational state is thought to facilitate recovery from infection and is characterized by a febrile response, reduced social activity, hyperalgesia, fatigue, adipsia and hypersomnia [4]. Contributions of IL-1β, TNF-α and IL-6 to LPS-induced sickness behaviour have been well described [5–7]. and direct comparisons of the effect of different cytokines on parameters such as temperature [8] and anorexia [9] have been performed. However, systematic comparisons of the effects on systemic and CNS inflammation of individual cytokines are lacking.

In recent years many researchers have implicated individual circulating cytokines as biomarkers of, or indeed contributors to, diverse clinical CNS conditions such as depression, delirium and dementia. Elevated IL-6 has been linked with reduced hippocampal volume and cognitive decline in the elderly [10–13] while studies of depression have implicated IL-1β, TNF-α and IL-6 [14,15]. Increased circulating pro-inflammatory cytokines have been associated with the neurodegenerative conditions of Alzheimer’s disease and Parkinson’s disease [16–18] and more specifically TNF-α has been linked with the accelerated progression of cognitive decline [19]. IL-6 and IL-8 (the functional homologue of CXCL1 in mouse) have been associated with delirium [20–23]. Despite these clinical studies implicating individual cytokines in CNS conditions, there is limited information regarding the direct impact of each cytokine on circulating and CNS markers of inflammation, and the effects of individual cytokines have not been systematically compared. There are many animal studies demonstrating deleterious CNS consequences of systemic inflammation, including depression-like [24,25] and delirium-like episodes [26] and exacerbation of neurodegenerative disease [27], but the gulf between clinical associations and understanding pathophysiological mechanisms remains wide. Systematic information on what CNS inflammatory mechanisms are initiated by the systemic elevation of individual cytokines constitutes an important resource in bridging this gulf.

The objective of the current study was to perform a systematic analysis of the effects of LPS (100 µg/kg), IL-1β (15 or 50 µg/kg), TNF-α (50 or 250 µg/kg) and IL-6 (50 or 125 µg/kg) in female C57BL6 mice. The effects of these inflammatory molecules on locomotor activity, core body temperature, plasma inflammatory mediators, as well as liver, hypothalamic and hippocampal gene transcription were compared 2 hours post-challenge, which we have previously identified as the peak of many of these responses to systemic LPS [28]. Given the more robust effects induced by systemic IL-1β and TNF-α challenges, the time course of the effects of 15 µg/kg IL-1β and 50 µg/kg TNF-α on peripheral and CNS inflammation was examined by sacrificing animals at 1, 2, 4, 8 and 24 hours after systemic injection. We also confirmed the physiological relevance of these mRNA changes by examining the expression of a smaller panel of inflammation-induced proteins in the CNS. The study provides the most comprehensive comparative analysis of the effects of systemic LPS, IL-1β, TNF-α and IL-6 on systemic and brain inflammation.

## Methods

### Animals

Female C57BL/6 mice (Harlan, Bicester, United Kingdom) were housed in groups of five at 21°C with a 12: 12 hour light dark cycle and given access to food and water *ad libitum*. Females were used in order to avoid fighting and injury in cages, which has a significant effect on behaviour. All animal experimentation was performed under a license granted by the Minister for Health and Children, Ireland, with approval from the Trinity College Dublin Animal Research Ethics Committee and in compliance with the Cruelty to Animals Act, 1876 and the European Community Directive, 86/609/EEC, and every effort was made to minimize stress to the animals.

### Intraperitoneal challenges

For behavioural and molecular experiments, C57BL/6 mice were injected with 100 µg/kg of the bacterial endotoxin LPS (equine abortus, Sigma, L5886, Poole, UK) prepared in non-pyrogenic 0.9% sterile saline (Sigma, Poole, UK), recombinant mouse IL-1β (R&D Systems, Minneapolis, MN, USA) at doses of 15 µg/kg or 50 µg/kg, recombinant mouse TNF-α (Peprotech, Rocky Hill, NJ, USA) at doses of 50 µg/kg or 250 µg/kg, recombinant mouse IL-6 (Peprotech, Rocky Hill, NJ, USA) at a dose of 50 µg/kg or 125 µg/kg or vehicle (0.9% sterile saline) in a volume of 200µl per 20g mouse. These doses were selected on the basis of a review of the literature on CNS effects of systemic cytokines (Table 1). We chose a relatively narrow range of concentrations in order to maximise the likelihood of measurable effects with both doses of each cytokine and, thus, to decrease animal use.

**Table 1:** Studies on which cytokine doses were based.

**Challenge**	**Reference**	**Dose**
IL-1β	Gosselin and Rivest, 2008 [59]	10 µg/kg
	Swiergiel and Dunn, 2007 [121]	5/15/50 µg/kg
	Elander et al., 2007 [122]	30 µg/kg
	Mormède et al., 2003 [123]	100 µg/kg
TNF-α	Wang et al., 1997 [8]	50 µg/kg
	Bluthe et al., 1994 [124]	125 µg/kg
IL-6	Wang et al., 1997; Wang and Dunn, 1998 [8,38]	50 µg/kg

### Sickness & behaviour

For all open field locomotor activity experiments, mice were moved from the housing room and left in the test room for 15 minutes prior to beginning the task to ensure an optimal state of arousal. The open field consisted of a plastic base (58 cm x 33cm) surrounded by walls of 19 cm. The floor of the box is divided into a grid of equal sized squares. Measurement was made of distance travelled (grid squares crossed) and total number of rears. Naïve animals had their baseline activity measured for 3 minutes when placed in the middle of the open field. At 2 hours post-challenge, the animals’ locomotor activity was measured again. A thermocouple rectal probe (Thermalert TH5, Physitemp, Clifton, NJ, USA) was used to measure core body temperature. The mice were pre-adapted to measurement of rectal temperature for 2 days prior to i.p. challenges to standardise stress effects. Temperatures were taken at baseline and then at 2 hours post-challenge.

### Plasma ELISA assays

Animals were terminally anaesthetised with sodium pentobarbital (40 mg per mouse i.p., Euthatal, Merial Animal Health, Essex, UK). The thoracic cavity was opened and blood collected in heparinised tubes directly from the right atrium of the heart. This whole blood was spun at 1.5 x *g* for 15 minutes to remove cells; the plasma was then aliquoted and stored at -20 °C. These samples were diluted appropriately and then analysed for IL-1β, TNF-α, IL-6, IL-1ra, CXCL1 (time course only), CCL2 (time course only), IFN-β, Corticosterone, and Prostaglandin E metabolites (PGEM).

Mouse IFNβ assay kit was supplied by Biosource (Nivelles, Belgium) and was performed as per manufacturers instructions. Mouse IL-1β, TNF-α, IL-6, IL-1ra, CCL2 and CXCL1 were quantified by sandwich-type ELISA, using R&D systems duo set kits (R&D systems, Minneapolis, MN, USA). A standard protocol was followed. The capture anti-IL-1β, TNF-α, IL-6, IL-1ra, CCL2 and CXCL1 antibodies were diluted 1/180 (approximately 1 µg/ml) in PBS, and used to coat a 96-well maxisorb microplate overnight (Nunc, Fisher Scientific, Leicestershire) with 100 µl per well. Plates were then washed with PBS + 0.05% Tween and blocked with PBS + 1% BSA (with 0.05% Tween 20 in Tris-buffered Saline for IL-1β) before addition of 100 µl samples and standards for 2 hours at room temperature. Detection antibodies were used at a dilution of 1/180 and incubated on the plates for 1.5 hours. Streptavidin poly-horseradish peroxidase (HRP: Sanquin, Amsterdam, Netherlands) was diluted in 1:10,000 and incubated in the dark at room temperature for 20 minutes. Samples, standards, detection antibodies and streptavidin poly-horseradish peroxidase were diluted in high performance ELISA buffer (Sanquin; Amsterdam, Netherlands). TMB and H_2_O_2_ were used as substrate and the reaction was stopped with 1 M H_2_S0_4_ before optical density was read at 450 nm with correction at 570 nm. Standard curves were constructed and samples were quantified only if the absorbance fell on the linear portion of the standard curve. Reliable quantification limits for the assays used were:

IL-1β 31.25 pg/ml, TNF-α 15.6 pg/ml, IL-6 15.6 pg/ml, IL-1ra 15.6 pg/ml, IFNβ 15.6 pg/ml, CXCL1 4 pg/ml, CCL2 4pg/ml.

Due to low levels of IL-1β expression in time course experiments, this cytokine was also analysed using a Quantikine mouse IL-1β kit (R&D systems, Minneapolis, MN, USA) according to manufacturers instructions.

### Corticosterone

The corticosterone ELISA was carried out using a kit supplied by Immunodiagnostic Systems (Tyne & Wear, UK) and the procedure for the competitive ELISA was carried out as per manufacturer’s instructions. Optical density was read at 450 nm with correction at 570 nm. Percent binding of calibrators, controls and samples were calculated.

### Prostaglandin E2 Metabolite

Plasma levels of prostaglandin E metabolites (PGEM) were measured using a PGEM EIA kit (Cayman Chemicals Ann Arbor, MI, USA). Prostaglandin E2 is rapidly converted *in-vivo* to its 13, 14-dihydro-15-keto metabolite, which is also chemically unstable and undergoes degradation to PGA products. Prostaglandin E Metabolite (PGEM) assay is a competitive assay that converts 13,14-dihydro-15-keto PGA_2_ and 13,14-dihydro-15-keto PGE_2_ to a single, stable derivative that can be quantified and used as a measure of initial PGE2 concentration. The assay is based on competition between sample PGEM and a PGEM-acetylcholinesterase (AChE) conjugate (PGEM tracer), for a limited number of PGEM-specific rabbit antiserum binding sites. Standards and samples were diluted in EIA buffer pre-derivitisation and PGEM buffer (EIA, carbonate buffer and phosphate buffer) post-derivitisation. Plasma samples and standards were derivatised by addition of carbonate buffer for overnight incubation at 37 °C, followed by addition of phosphate buffer and EIA buffer. 100 µl diluted plasma samples, calibrators and controls were added to the plate pre-coated with mouse anti-rabbit IgG. Controls were as follows, blank, total activity, non-specific binding and maximum binding. PGEM-acetylcholinesterase conjugate was added to all wells except total activity and blank. Specific rabbit antiserum to PGEM was added to all wells except total activity, non-specific binding and blank. The plate was then covered and incubated for 18 hours at room temperature. The following day, the plate was washed 5 times with wash buffer before Ellman’s reagent was added to all wells; this assay was developed for 80 minutes and optical density was read at 420 nm. Non-specific binding was subtracted from maximum binding, and then percent binding of standards and samples were calculated.

### RNA extraction, reverse transcription cDNA synthesis and quantitative PCR

Animals were transcardially perfused with heparinised saline, as described above, and brains were rapidly removed, and the meninges removed from them. Hippocampi and hypothalami were dissected out, without attention to the removal of the circumventricular organs, placed in eppendorf tubes, snap frozen on liquid nitrogen and stored at -80 °C until further use. Liver samples were also taken and treated in a similar manner. Total RNA was extracted from brain and liver samples using Qiagen RNeasy Plus™ mini kits (Qiagen, Crawley, UK: all 2 hour data) or Macherey-Nagel Nucleospin® RNA II kits (Macherey-Nagel, Düren, Germany: time course data), in accordance with manufacturer’s instructions. Samples were weighed to ensure each lay in the range of 20–40 mg. RNA yields and purity were determined by spectrophotometry at 260 and 280 nm using the NanoDrop ND-1000 UV–Vis Spectrophotometer (Thermo, Fisher Scientific, Dublin, Ireland). Quantified RNA was stored at −80°C until cDNA synthesis and PCR assay.

Using a High Capacity cDNA Reverse Transcriptase Kit (Applied Biosystems, Warrington, UK), cDNA was synthesised from total RNA. 400 ng of total RNA were reverse transcribed per 20 µl reaction volume and 1 µl of the reverse transcription reaction (RT) was used for polymerase chain reaction (PCR). For a 20 µl volume reaction, 10 µl master mix (containing: 2 µl 10X RT Buffer; 0.8 µl 25X dNTP mix, 100mM; 2 µl 10X RT random primers; 1 µl MultiScribe™ Reverse Transcriptase; 4.2 µl RNase-free water) was added to 10 µl RNA + RNAse-free water in a nuclease-free PCR tube (Greiner Bio-One, Monroe, NC, USA). No reverse transcriptase and no RNA template controls were also prepared. PCR tubes were placed in a DNA Engine® Peltier Thermal Cycler PTC-200 (Bio-Rad Laboratories, Inc., Hercules, CA, USA) and samples were incubated at 25°C for 10 minutes, 37°C for 120 minutes and 85°C for 5 seconds. Samples were held at 4°C until collection and then stored at −20°C until assay.

The StepOne™ Real-Time PCR system was supplied by Applied Biosystems (Warrington, UK) and used in 96-well format with a 25µl reaction volume per well for quantitative (Q)-PCR. In general, forty cycles were run with the following conditions: 2 minutes at 50^°^C, 10 minutes at 95^°^C and for each cycle 15 seconds at 95^°^C to denature and 1 minute at 60^°^C for transcription. Reagents were supplied by Applied Biosystems (SYBR Green PCR Master Mix) and Roche (FastStart Universal Probe Master [Rox] and FastStart Universal SYBR Green Master [Rox], Lewes, UK). Assays were designed using the published sequences for the genes of interest and Primer Express software (Applied Biosystems, Warrington, UK). Primer and probe sequences are shown in Table 2. Where possible, probes were designed to cross introns such that they were cDNA specific. In some cases the fluorescent DNA binding probe SYBR green was used in place of a specific probe. All primer pairs were checked for specificity by standard reverse transcription (RT)-PCR followed by gel electrophoresis and each primer pair produced a discrete band of the expected amplicon size. Assays were quantified using a relative standard curve as previously described [29]. All PCR data were normalised to the expression of the housekeeping gene glyceraldehyde-3-phosphate dehyrdrogenase (GAPDH).

**Table 2:** Quantitative PCR primer and probe sequences.

**Target**	**Accession no.**	**Oligo**	**Sequence**	**Amplicon** **size (bp)**
CCL2	NM_011333	Forward	5’-GTTGGCTCAGCCAGATGCA-3’	81
		Reverse	5’-AGCCTACTCATTGGGATCATCTTG-3’	
		Probe	5’-TTAACGCCCCACTCACCTGCTGCTACT-3’	
CD11b	NM_001082960.1	Forward	5’-TCATTCGCTACGTAATTGGG-3’	90
		Reverse	5’-GATGGTGTCGAGCTCTCTGC-3’	
COX-1	NM_008969	Forward	5’-CCAGAACCAGGGTGTCTGTGT-3’	70
		Reverse	5’-GTAGCCCGTGCGAG TACA ATC-3’	
		Probe	5’-CGCTTTGGCCTCGACAACTACCAGTG-3’	
COX-2	NW_000157	Forward	5’-GAGTGGTAGCCAGCAAAGCC-3’	81
		Reverse	5’- TTTAATTGGGAACCCTTCTTTGTT-3’	
		Probe	5’-AGCAACAAAAGCGTTCTACAAAGGAACTAACCA-3	
CXCL1	NM_008176	Forward	5’-CACCCAAACCGAAGTCATAGC-3’	82
		Reverse	5’-AATTTTCTGAACCAAGGGAGCTT-3’	
GAPDH	NM_008084.2	Forward	5’-GACGGCCGCATCTTCTTGT-3’	65
		Reverse	5’-CAGTGCCAGCCTCGTCCCGTAGA-3’	
IFN-α	BX_530016	Forward	5’-TCTGATGCAGCAGGTGGG-3’	159
		Reverse	5’-AGGGCTCTCCAGACTTCTGCTCTG-3’	
IFN-β	X14455	Forward	5’-CCATCATGAACAACAGGTGGAT-3’	67
		Reverse	5’-GAGAGGGCTGTGGTGGAGAA-3’	
		Probe	5’-CTCCACGCTGCGTTCCTGCTGTG-3’	
IL-1β	M15131	Forward	5’-GCACACCCACCCTGCA-3’	69
		Reverse	5’-ACCGCTTTTCCATCTTCTTCTT-3’	
		Probe	5’-TGGAGAGTCTGGATCCCAAGCAATACCC-3’	
IL-6	NM_031168	Forward	5’-TCCAGAAACCGCTATGAAGTTC-3’	72
		Reverse	5’-CACCAGCATCAGTCCCAAGA-3’	
		Probe	5’-CTCTGCAAGAGACTTCCATCCAGTTGCC-3’	
PTX3	X_83601	Forward	5’-ACAACGAAATAGACAATGGACTTCAT-3’	69
		Reverse	5’-CTGGCGGCAGTCGCA-3’	
SAA	M13522	Forward	5’-GCCATGGAGGGTTTTTTTCATT-3’	80
		Reverse	5’-CCTTTGGGCAGCATCATAGTTC-3’	
		Probe	5’-CACATGTCTCCAGCCCCTTGGAAAGC-3’	
TNF-α	M11731	Forward	5’-CTCCAGGCGGTGCCTATG-3’	149
		Reverse	5’-GGGCCATAGAACTGATGAGAGG-3’	
		Probe	5’-TCAGCCTCTTCTCATTCCTGCTTGTGG-3’	
TNF-αIP2	NM_009396	Forward	5’-GCTGGCAAGGCACATCCT-3’	74
		Reverse	5’-AGGTTGCAGTGGAGCCATTCT-3’	
		Probe	5’-CAACGCGGATGCC-3’	
uPAR	NM_011113.3	Forward	5’-TGCAATGCCGCTATCCTACA-3’	116
		Reverse	5’-TGGGCATCCGGGAAGACT-3’	
		Probe	5’-CCCTCCAGAGCACAGAAAGGAGCTTGAA-3’	
VCAM	NM_011693.3	Forward	5’-GATGTAAAAGGAAAAGAACATAACAAGAAC-3’		
		Reverse	5’-GATGGCAGGTATTACCAAGGAAGA-3’ 90	

### Immunohistochemistry

Animals were transcardially perfused with heparinised saline followed by 10% neutral buffered formalin (Sigma, Poole, UK) for approximately 15 minutes/50 mls of fixative. Brains were then removed and post-fixed in the same fixative for 3 days before embedding in paraffin wax. Coronal sections (10 µm) of brains were cut on a Leica RM2235 Rotary Microtome (Leica Microsystems, Wetzlar, Germany) at the various positions along the anterior–posterior axis from Bregma, including hypothalamus, amygdala and hippocampus and floated onto electrostatically charged slides (Menzel-Glaser, Braunschweig, Germany) and dried at 37°C overnight. All sections were quenched for 20 minutes in 1% hydrogen peroxide in methanol, microwaved in citrate buffer (pH 6) for 2x5 minutes and blocked in 10% normal serum for 1h (2h for COX-2 labeling). Additional treatments with 1% triton for 15 minutes for p65 and cFOS labeling, and 0.04% pepsin in 0.01M HCl for 20 minutes for CCL2 labeling were performed prior to blocking. Primary antibodies were used as follows: mouse anti-p65 (1/1000, Santa Cruz), goat anti-COX2 (1/1000, Santa Cruz), goat anti-CCL2 capture antibody (1/1000, R&D Systems CCL2 duo-set ELISA antibody pair), goat anti-CXCL1 (1/50, R&D Systems), mouse anti-cFOS (1/250, Santa Cruz), rabbit anti-IL-1β (1/50 in 20% normal goat serum, Peprotech) and goat anti-TNF-α capture antibody (1/1000, R&D Systems ELISA kit). They were incubated overnight at 4^o^C. Sections were then incubated in appropriate biotinylated secondary antibody at 1/100 (Vector Labs) and developed using the ABC method with hydrogen peroxide and diaminobenzidine (DAB) as substrate.

### Statistics

For all 2 hour measures, experimental groups were analysed using one-way ANOVA. Where a significant main effect was found selected pairwise comparisons were made using Bonferroni post-hoc analysis. In all cases, the high dose of each pro-inflammatory cytokine was compared to saline/LPS. In addition, where there was not a significant difference between high dose cytokine and saline and the low dose mean was greater than the high dose, a comparison between low does and saline/LPS was made. Time course data were analysed by two-way ANOVA with time as within subjects factor and treatment as between subjects factor. Separate pairwise analysis has been performed to compare high dose to low dose challenge.

## Results

### Sickness behaviour

**Figure 1:**
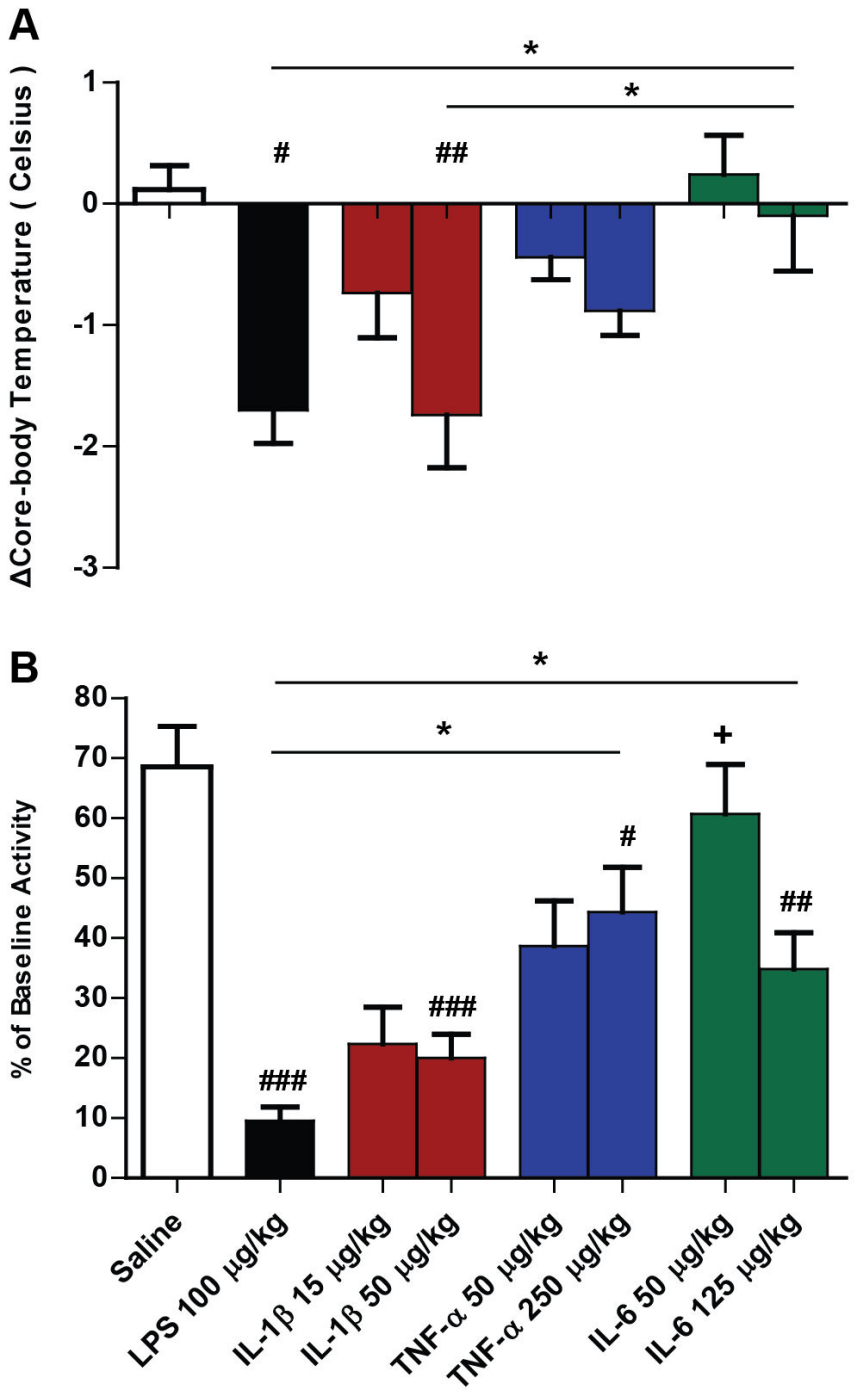
Impact of systemic LPS, IL-1β, TNF-α and IL-6 treatment on core body temperature and locomotor activity. Deviation in core-body temperature from baseline (a) and in locomotor activity (as a percentage of baseline activity at t=0) (b) were assessed 2 hours after systemic challenge (i.p.) with saline, LPS (100 µg/kg), IL-1β (15 µg/kg or 50 µg/kg), TNF-α (50 µg/kg or 250 µg/kg) and IL-6 (50 µg/kg or 125 µg/kg). Data were analysed by one-way ANOVA, followed by Bonferroni post-hoc tests comparing differences between saline, LPS and each cytokine treatment at the higher dose. # denotes treatment is significantly different to saline control ( # p<0.05 # # p<0.01, # # # p<0.001). * denotes differences between treatments p < 0.05 (n=5 for all groups, except LPS n=4 and 15 µg/kg IL-1β n=3). + denotes significant difference between low and high dose of cytokine p<0.05. All data have been presented as mean ± SEM.

The effect of systemic treatment (i.p.) with saline, LPS (100 µg/kg), IL-1β (15 µg/kg or 50 µg/kg), TNF-α (50 µg/kg or 250 µg/kg) and IL-6 (50 µg/kg or 125 µg/kg) on the deviation of core body temperature from baseline (t = 0) at 2 hours post-challenge in C57BL/6 female mice was examined (Figure 1a). A Bonferroni post-hoc test following a significant one-way ANOVA (F = 6.552, df 4,19, p = 0.0017) revealed that injection of LPS and IL-1β induced marked hypothermic responses with respect to both saline and IL-6 challenges (p<0.05). TNF-α induced a one-degree decrease in temperature but this was not statistically significant (p> 0.05) in comparison with saline treatment. IL-6 did not produce any change in core body temperature (p>0.05).

Systemic LPS, IL-1β, TNF-α and IL-6 induced a clear reduction in locomotor activity 2 hours post-challenge, in comparison with saline-challenged (Figure 1b). These differences were shown by a Bonferroni post-hoc test (p<0.05) following a significant one-way ANOVA (F=16.34, df 4,20, p=0.0001). LPS treated animals were significantly less active than the TNF-α (p<0.05) and IL-6-treated animals (p<0.05). IL-6 low dose and high dose-treated animals also differed significantly from each other in terms of reduction in locomotor activity (p<0.05).

### Plasma inflammatory mediators

**Figure 2:**
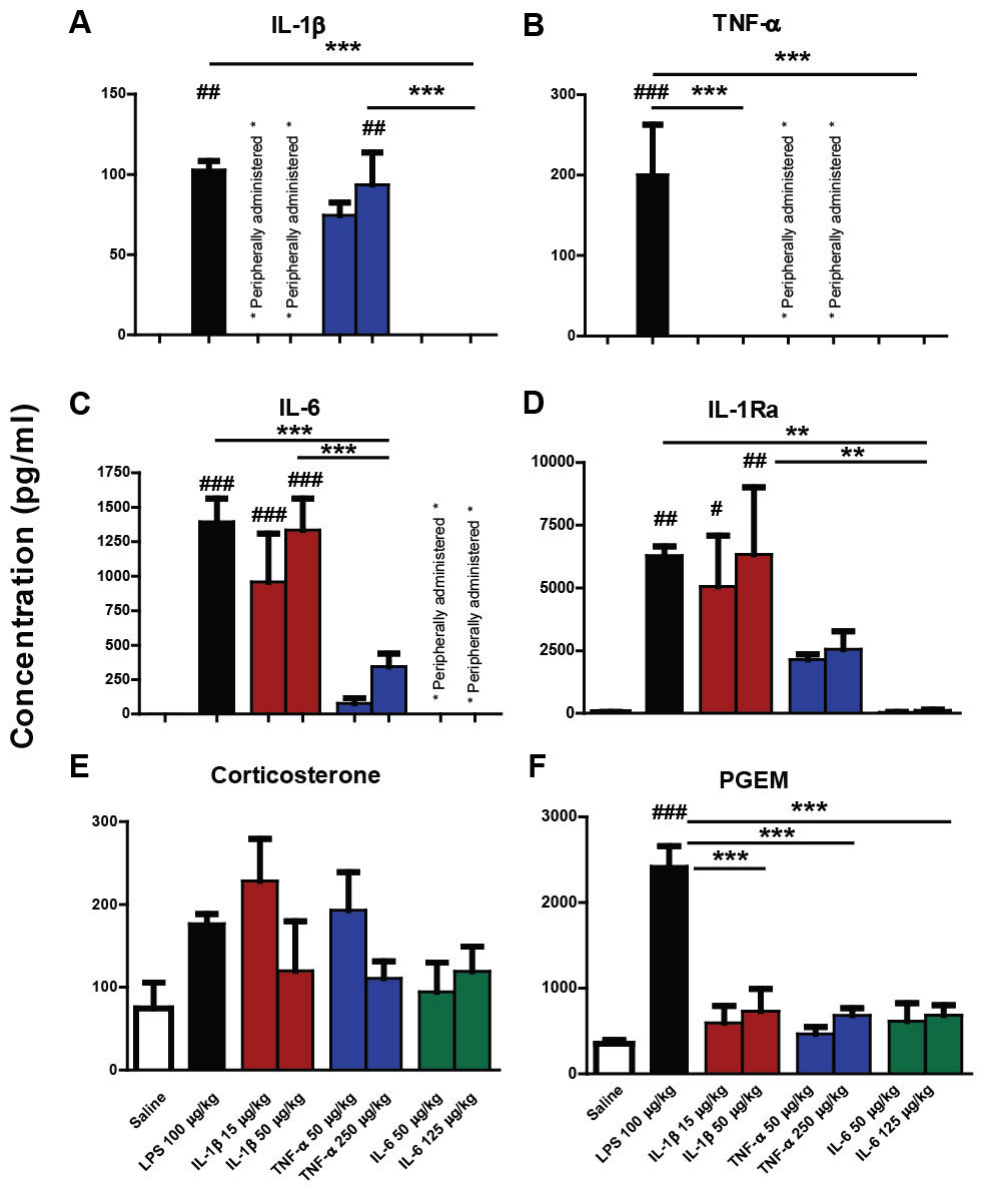
Impact of systemic LPS, IL-1β, TNF-α and IL-6 challenge on plasma inflammatory and stress mediators. IL-1β (a), TNF-α (b), IL-6 (c), IL-1ra (d), corticosterone (e) and PGEM (f) as measured by ELISA in plasma prepared from whole blood of C57BL/6 female mice 2 hours after systemic challenge (i.p.) with saline, LPS (100 µg/kg), IL-1β (15 µg/kg or 50 µg/kg), TNF-α (50 µg/kg or 250 µg/kg) or IL-6 (50 µg/kg or 125 µg/kg). All data groups were compared by one-way ANOVA, followed by Bonferroni post-hoc tests comparing differences between saline, LPS and each cytokine treatment at the higher dose. Endogenous versus injected levels could not be discriminated for IL-1β, TNF-α or IL-6 (denoted peripherally administered). # denotes treatment is significantly different to saline control # p<0.05. ** denotes significant difference between treatment groups indicated by line p < 0.01, ***p < 0.001. All data have been presented as mean ± SEM. n=5 for all groups except LPS and IL-1β 50 µg/kg n=4 and IL-1β 15 µg/kg n=3.

To assess the impact of the peripheral immune challenges on pro-inflammatory cytokines in the plasma of mice, ELISAs were performed for IL-1β, TNF-α, IL-6, IFN-β on blood collected 2 hours post-administration (Figure 2). The appearance of the injected cytokine in the plasma was confirmed for each of the three cytokines used. The results obtained for IL-1β levels in IL-1β-treated animals, for TNF-α in TNF-α-treated animals and IL-6 levels in IL-6-treated animals were excluded from the analysis since injected and induced cytokine could not be distinguished. However, they are reported and compared to their LPS-induced levels in Table 3. A significant one-way ANOVA (F=13.24, df 3,15, p=0.002) with Bonferroni pairwise tests for levels of circulating IL-1β revealed LPS and TNF-α treatments to be significantly different from saline (at detection limit) and IL-6 (at detection limit) (p<0.01). Thus, IL-1β was induced by TNF-α but not by IL-6. IL-1β levels were approximately 5-fold higher after 50µg/kg compared to 15 µg/kg, and this lower doses resulted in IL-1β levels 1-2 fold higher than that induced by LPS at 100 µg/kg (Table 3).

**Table 3:** Plasma Cytokine levels 2 hours post-cytokine or LPS injection.

Treatment	Serum level of injected cytokine (pg/mL)	Serum levels generated by 100µg/kg LPS (pg/mL)
IL-1β (15µg/kg)	284 ±155	125±40
IL-1β (50µg/kg)	1309±297	
TNF-α (50µg/kg)	269±77	200±72
TNF-α (250µg/kg)	7077±388	
IL-6 (50µg/kg)	nd	
IL-6 (125µg/kg)	138±15	1393±194

Plasma levels of cytokines 2h after their own i p administration were measured and compared to LPS-induced levels. Data presented represent mean±S.E.M from 5 animals. nd: not detectable.

TNF-α was robustly induced by LPS challenge but undetectable in IL-1β- and IL-6-treated animals. Bonferroni post-hoc comparisons after a significant one-way ANOVA (F=11.48, df 4,15, p=0.0004) revealed these levels to be greater than those induced by saline and IL-6, which were at the detection limit of the assay (p<0.01). There was no significant difference between saline, IL-6- and IL-1β-treated animals (p > 0.05). Therefore, IL-6 and IL-1β administration did not induce systemic TNF-α at 2 hours. TNF-α levels were approximately 25-fold higher after 250 µg/kg TNF-α than after 50µg/kg TNF-α, and these lower levels were very similar to levels induced by LPS (Table 3).

A one-way ANOVA (F=13.24, df 3,15, p=0.002) with Bonferroni pairwise tests for circulating IL-6 revealed that LPS and IL-1β induced significantly greater levels of IL-6 than those induced by saline and TNF-α (p<0.0001). IL-6 levels were not elevated significantly in IL-6 animals compared to saline (p<0.05), in contrast to IL-1β and TNF-α induction after concomitant challenges. In summary, IL-6 was increased by IL-1β administration but not TNF-α or IL-6 at 2 hours post-challenge. IL-6 appeared to be rapidly cleared, since its levels 2 hours after injection of 50µg/kg were undetectable and those 2 hours after injection of 125 µg/kg at levels were 10 fold less than that induced by LPS (Table 3).

Although low in comparison to levels induced by poly I:C challenge [30] IFN-β was clearly induced after LPS treatment (26.45±6.41 pg/mL), However, IFN-β was not detected after saline, IL-1β, TNF-α or IL-6 treatments. Thus only LPS-challenge induced detectable plasma IFN-β.

We also evaluated blood levels of IL-1ra (Figure 2d). LPS and both doses of IL-1β increased IL-1ra (F=6.500, df 7,29, p<0.05). No significant differences could be observed between high and low dose of IL-1β, nor for TNF-α-induced expression, although the latter clearly induced IL-1ra.

Corticosterone and prostaglandin E metabolite (PGEM; measured as a proxy for rapidly metabolised plasma levels of PGE2) levels were also assessed in blood collected 2 hours post-challenge (Figure 2e-f). Following a statistically significant one-way ANOVA (F=3.031, df 4,17, p=0.0467), Bonferroni post-hoc pairwise comparisons revealed no significant differences in circulating corticosterone levels between the experimental groups (p>0.05). Due to significant variability we cannot state that corticosterone levels are significantly elevated, but LPS, IL-1β and TNF-α all showed a strong trend towards corticosterone induction that was not quite significant. One-way ANOVA (F=21.57, df 4,16, p=0.0001) followed by Bonferroni post-hoc comparisons showed that the LPS treatment group was significantly different to all other treatment groups for PGEM (p<0.001). While the higher dose of TNF-α, IL-6 and IL-1β induced some small non-significant increases in PGEM at 2 hours (p>0.05), only LPS treatment resulted in robustly elevated circulating PGEM.

### Liver mRNA transcription

**Figure 3:**
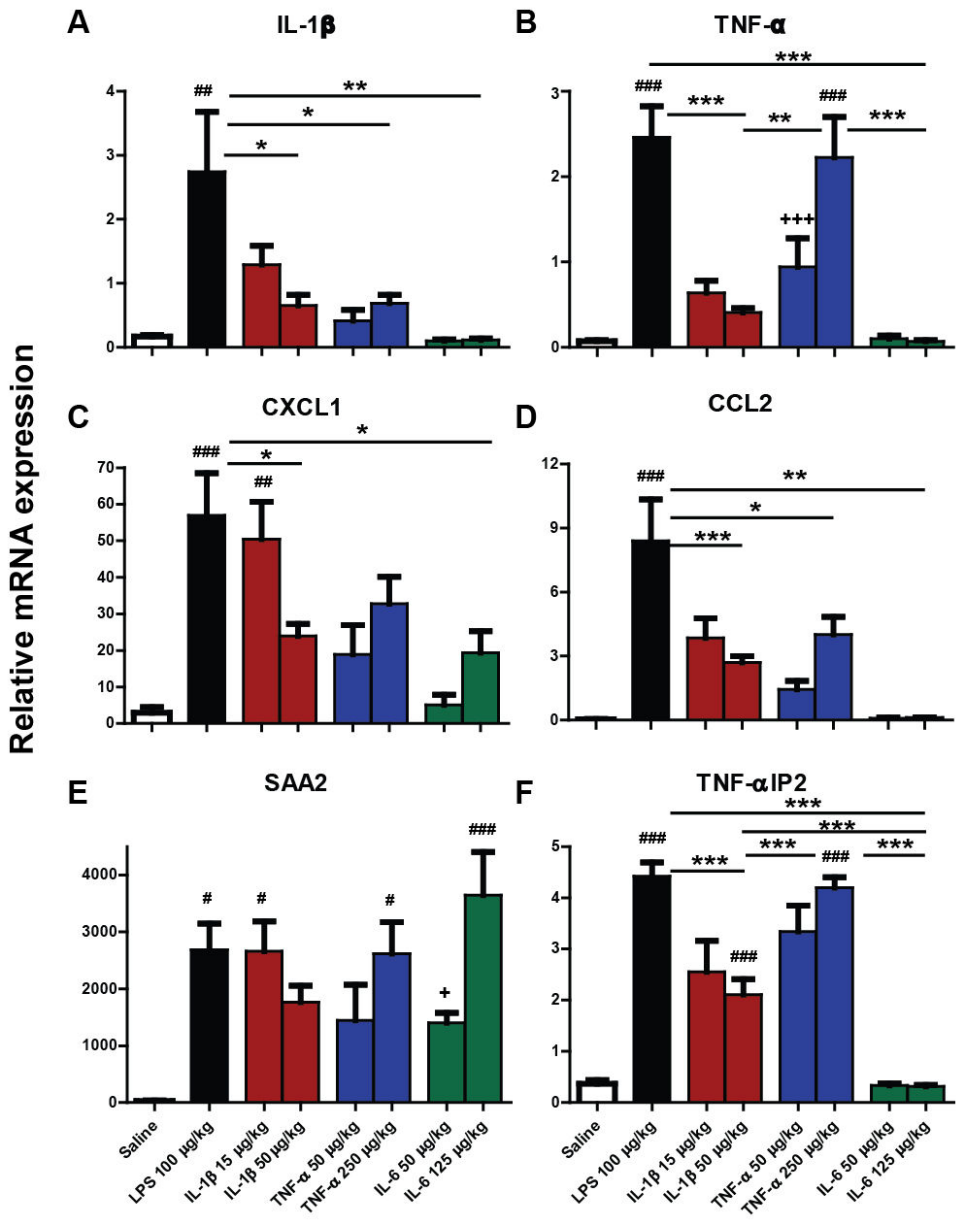
Impact of systemic LPS, IL-1β, TNF-α and IL-6 challenge on mRNA transcription of hepatic pro-inflammatory cytokines IL-1β, TNF-α and downstream genes. Liver transcription of mRNA species for IL-1β, TNF-α, CXCL1, CCL2, SAA2 and TNF-αIP2 were measured using quantitative PCR in C57BL/6 female mice 2 hours after systemic challenge (i.p.) with saline, LPS (100 µg/kg), IL-1β (15 µg/kg or 50 µg/kg), TNF-α (50 µg/kg or 250 µg/kg) and IL-6 (50 µg/kg or 125 µg/kg). Data were analysed by one-way ANOVA, followed by Bonferroni post-hoc tests comparing differences between saline, LPS and each cytokine treatment at the higher dose. ##, denotes treatment is significantly different to saline treatment # # p<0.01, # # # p<0.001. * denotes significant difference between treatment groups indicated by line p < 0.05, **p<0.01, ***p<0.001. +++ denotes significant difference between low and high dose of cytokine p<0.001. All data have been presented as mean ± SEM, n=5 for all groups.

To assess the hepatic inflammatory response in animals 2 hours post-peripheral challenge, liver mRNA transcription of pro-inflammatory cytokines IL-1β and TNF-α, acute phase protein CXCL1, CCL2 and serum amyloid alpha 2 (SAA2), as well as the downstream gene TNF-αIP2 was measured using quantitative PCR (Figure 3).

Hepatic IL-1β mRNA was induced to significantly higher levels by peripheral LPS-treatment than IL-1β, IL-6 and TNF-α-treatment (p<0.05). This was demonstrated by a significant one-way ANOVA (F=6.270, df 4,20, p=0.0019), followed by Bonferroni comparisons (p<0.05). None of the pro-inflammatory cytokines induced statistically significant transcription of IL-1β mRNA, although both IL-1β and TNF-α showed trends.

TNF-α was significantly induced in LPS-treated and TNF-α-treated animals, compared to saline-treated animals. TNF-α low and high dose-treatment groups also differed significantly by Bonferroni post-hoc tests (p<0.05) after a significant one-way ANOVA (F=14.50, df 5,24, p=0.0001).

Bonferroni post-hoc comparisons after a significant one-way ANOVA (F=7.453, df 4,20, p=0.0008) showed that CXCL1 was significantly induced in LPS and low dose IL-1β-treated animals compared to saline (p<0.05). LPS treatment induced significantly higher CXCL1 mRNA levels than high dose IL-1β and IL-6 (p<0.01). Thus, peripheral injection of the low dose of IL-1β induced higher hepatic CXCL1 mRNA levels than injection of the high dose and the levels elicited were almost equivalent to those brought about by LPS challenge. TNF-α administration induced more than a ten-fold increase in CXCL1 mRNA. Moreover, IL-6 treatment also induced a considerable fold change in transcription. However, both of these increases were not statistically significant in comparison to saline challenge (p>0.05).

LPS treatment induced significantly more hepatic CCL2 mRNA than saline challenge and challenge with any pro-inflammatory cytokine. Nonetheless CCL2 was significantly induced by both IL-1β and TNF-α, but not IL-6, as revealed by a significant one-way ANOVA (F=12.87, df 4,20, p=0.0001) with Bonferroni comparisons (p<0.05).

Peripheral challenge with IL-6 elicited a robust increase in SAA2 (Figure 3E). Other than eliciting a reduction in open field activity, this is the only statistically significant evidence of IL-6 induced-biological activity in the current study. Following a significant one-way ANOVA (F=7.858, df 4,20, p=0.0006), post-hoc tests showed that LPS, IL-1β, TNF-α and IL-6 significantly induced SAA2 mRNA transcription in comparison to saline challenge. IL-6 low dose and high dose also differed significantly (p<0.05).

TNF-αIP2, a gene described as being expressed downstream of TNF-α [31], was actually robustly induced by LPS, IL-1β and TNF-α. Following a significant one-way ANOVA (F=95.93, df 4,20, p=0.0001), Bonferroni post-hoc comparison revealed that LPS, IL-1β and TNF-α challenges produced significantly higher levels of TNF-αIP2 than saline-challenges. Levels elicited by LPS and high dose TNF-α were significantly higher than the high dose of IL-1β (all p<0.0001). IL-6 did not induce TNF-αIP2.

### Hypothalamic mRNA transcription

**Figure 4:**
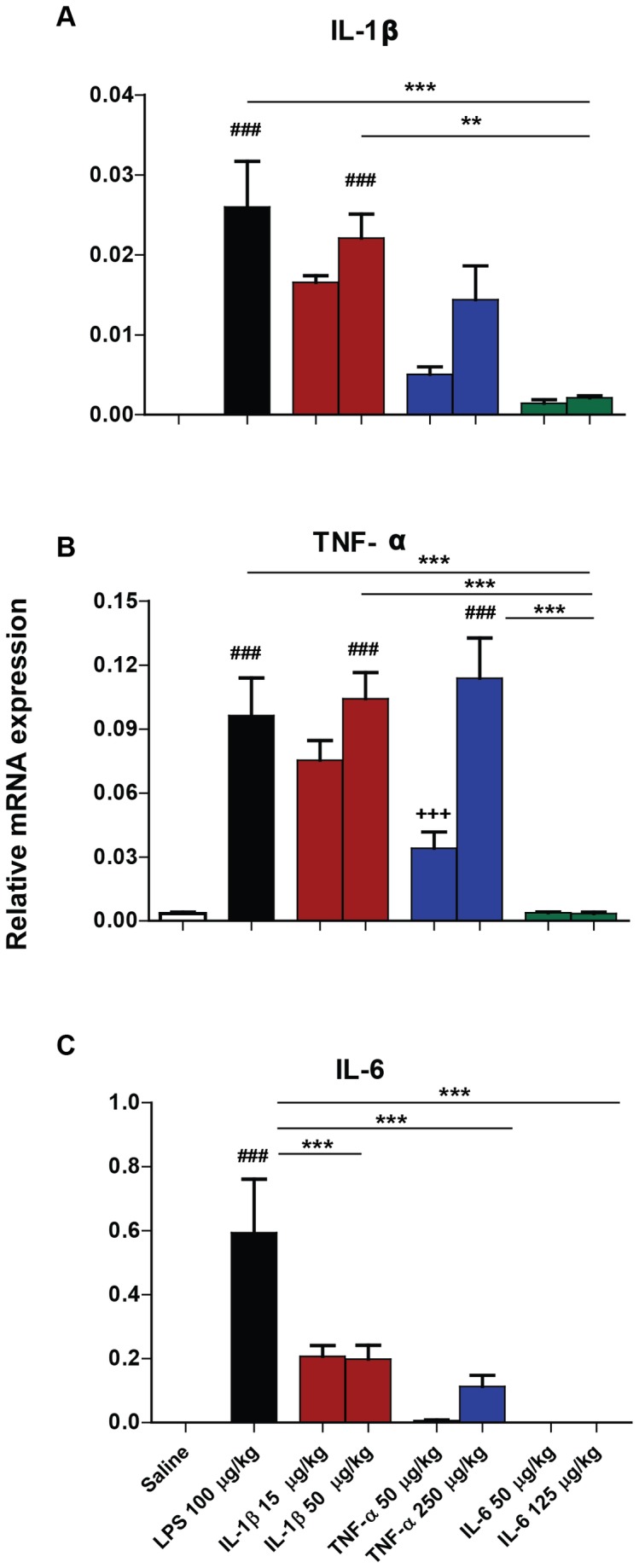
Impact of systemic LPS, IL-1β, TNF-α and IL-6 challenge on hypothalamic mRNA transcription. Hypothalamic transcription of mRNA species for IL-1β, TNF-α and IL-6 assessed using quantitative PCR 2 hours after systemic challenge (i.p.) with saline, LPS (100 µg/kg), IL-1β (15 µg/kg or 50 µg/kg), TNF-α (50 µg/kg or 250 µg/kg) and IL-6 (50 µg/kg or 125 µg/kg). Data were analysed by one-way ANOVA, followed by Bonferroni post-hoc tests comparing differences between saline, LPS and each cytokine treatment at the higher dose. ## denotes treatment is significantly different to saline treatment # # p<0.01, # # # p<0.001. ** denotes significant difference between treatment groups indicated by line p < 0.01, *** p<0.001. All data have been presented as mean ± SEM, n=5 for all groups, except IL-1β 15 µg/kg n=3.

Hypothalamic mRNA transcription of pro-inflammatory cytokines IL-1β, TNF-α and IL-6 was measured (Figure 4) using quantitative PCR in animals 2 hours post-peripheral challenge with saline, LPS (100 µg/kg), IL-1β (15 µg/kg or 50 µg/kg), TNF-α (50 µg/kg or 250 µg/kg) and IL-6 (50 µg/kg or 125 µg/kg).

LPS and IL-1β challenges induced significant transcription of IL-1β mRNA compared to saline and IL-6 challenges, demonstrated by Bonferroni post-hoc comparisons (p<0.01) following a significant one way ANOVA (F=11.38, df 4,20, p=0.0001). There was a trend towards a TNF-α induced increase in IL-1β mRNA but this was not significantly different to saline-treated animals (p>0.05).

Hypothalamic TNF-α mRNA was induced robustly by systemic LPS, IL-1β and TNF-α challenge compared to both saline and IL-6. TNF-α low dose and high dose also differed significantly. These differences were shown by Bonferroni post-hoc tests (p<0.001) after a significant one way ANOVA (F=18.22, df 5,22, p=0.0001). Thus, peripheral administration of both TNF-α and IL-1β elicited marked hypothalamic TNF-α mRNA, as did LPS. However, IL-6 administration had no effect on mRNA levels, when compared to control treatment.

Hypothalamic IL-6 mRNA was significantly up-regulated solely by LPS challenge. A significant one-way ANOVA (F= 8.897, df 4,19, p=0.0003) followed by Bonferroni post-hoc tests shows that this LPS-induced increase in IL-6 is significant compared to saline, IL-1β, TNF-α and IL-6 challenges (p<0.001). When LPS treatment is excluded from the analysis, IL-1β has a significant effect on IL-6 mRNA (p<0.01) in comparison with saline and IL-6 administration.

### Hippocampal expression of inflammatory transcripts

**Figure 5:**
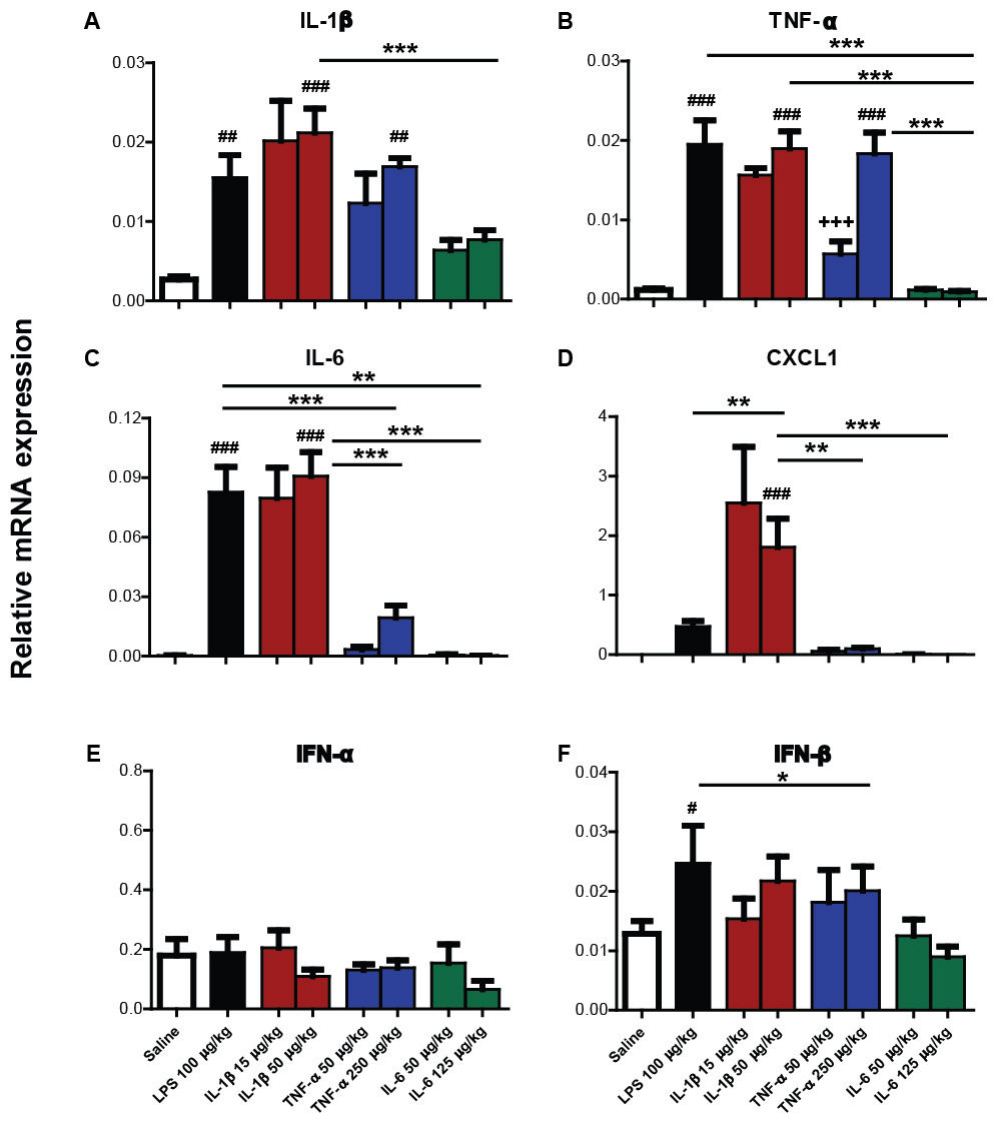
Impact of systemic LPS, IL-1β, TNF-α and IL-6 challenge on hippocampal inflammatory cytokine transcription. Hippocampal transcription of IL-1β, CXCL1, IL-6, TNF-α, IFN-α and IFN-β mRNA measured using quantitative PCR 2 hours after systemic challenge (i.p.) with saline, LPS (100 µg/kg), IL-1β (15 µg/kg or 50 µg/kg), TNF-α (50 µg/kg or 250 µg/kg) or IL-6 (50 µg/kg or 125 µg/kg). All data groups were compared by one-way ANOVA, followed by Bonferroni post-hoc tests comparing differences between saline, LPS and each cytokine treatment at the higher dose. ## denotes treatment is significantly different to saline treatment # # p<0.01, # # # p<0.001. ** denotes significant difference between treatment groups indicated by line p < 0.01 *** p<0.001. +++ denotes significant difference between low and high dose of cytokine p<0.001. All data have been presented as mean±SEM, n=5 for LPS and IL-1β 50 µg/kg; n≥4 for TNF-α 250 µg/kg; n≥3 for other groups.

Hippocampal mRNA transcription of pro-inflammatory cytokines IL-1β, CXCL1, IL-6, TNF-α, IFN-α and IFN-β was measured (Figure 5) using quantitative PCR in animals 2 hours post-peripheral challenge with saline, LPS (100 µg/kg), IL-1β (15 µg/kg or 50 µg/kg), TNF-α (50 µg/kg or 250 µg/kg) and IL-6 (50 µg/kg or 125 µg/kg). Hippocampal IL-1β mRNA was significantly induced by peripheral administration of LPS, IL-1β and TNF-α in comparison to saline control challenge as demonstrated by Bonferroni post-hoc test (p<0.005) after a significant one-way ANOVA (F=11.41, df 43,18, p<0.0001). IL-1β challenge also induced significantly higher levels than IL-6 (125 µg/kg) (p<0.001).

TNF-α mRNA was significantly upregulated by systemic LPS, IL-1β and TNF-α challenge compared to both saline and IL-6. TNF-α low dose and high dose also differed significantly. These differences were shown by Bonferroni post-hoc tests (p<0.001) after a significant one way ANOVA (F=22.67, df 5,23, p<0.0001).

IL-6 mRNA was induced robustly by systemic LPS and IL-1β in comparison with saline, TNF-α and IL-6 (125 µg/kg) challenge, shown by Bonferroni post-hoc tests (p<0.001), after a significant one-way ANOVA (F=20.81, df 4,16, p=0.0001). TNF-α and IL-6 administration did not induce IL-6 mRNA levels that were different to saline challenge (p>0.05). Thus, neither peripheral administration of TNF-α nor IL-6 induces hippocampal IL-6 transcription at 2 hours post-challenge.

Hippocampal CXCL1 mRNA was significantly upregulated solely by IL-1β (50 µg/kg) challenge. A significant one-way ANOVA (F=10.47, df 4,18, p<0.0001) followed by Bonferroni post-hoc tests shows that this IL-1β-induced increase in CXCL1 was significantly greater than saline, LPS, TNF-α and IL-6 challenges (p<0.01).

IFN-α mRNA was not significantly altered by systemic challenge (F=1.292, df 4,18, p=0.3101). IFN-β mRNA, however, was significantly induced by LPS administration. A one-way ANOVA (F=4.454, df 3,16, p=0.0131) and Bonferroni post-hoc test showed that LPS treatment was significantly greater than saline, TNF-α and IL-6 (125 µg/kg) (p<0.05).

**Figure 6:**
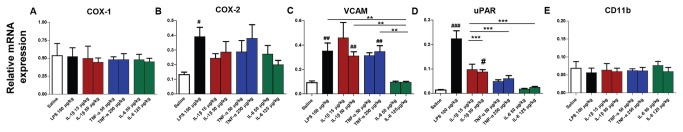
Impact of systemic LPS, IL-1β, TNF-α and IL-6 challenge on cyclooxygenases and cell markers. Hippocampal transcription of the biosynthetic enzymes COX-1, COX-2, VCAM, uPAR and CD11b mRNA measured using quantitative PCR in C57BL/6 female mice 2 hours after systemic challenge (i.p.) with saline, LPS (100 µg/kg), IL-1β (15 µg/kg or 50 µg/kg), TNF-α (50 µg/kg or 250 µg/kg) and IL-6 (50 µg/kg or 125 µg/kg). All data groups were compared by one-way ANOVA, followed by Bonferroni post-hoc tests comparing differences between saline, LPS and each cytokine treatment at the higher dose. # denotes treatment is significantly different to saline treatment p<0.05, # # p<0.01, # # # p<0.001. *** denotes significant difference between treatment groups indicated by line p < 0.001. All data have been presented as mean±SEM, n=5 for all groups except n=4 for TNF-α 250 µg/kg; n≥3 for IL-1β 15 µg/kg and both IL-6 groups.

The mRNA transcription of prostaglandin biosynthetic enzymes COX-1 and COX-2, and the endothelial marker VCAM, microglial and putative endothelial marker uPAR [32] and microglial activation marker CD11b were examined after the systemic pro-inflammatory challenges (Figure 6). COX-1 mRNA was not significantly altered by treatment as shown by a one-way ANOVA (F=0.1262, df 4,18, p=0.9710) while COX-2 mRNA was significantly altered by treatment (one-way ANOVA: F=3.688, df 4,19, p=0.0219). Bonferroni post-hoc test showed that LPS treatment induced COX-2 to a significantly greater degree than saline (p<0.05). The pro-inflammatory cytokines IL-1β and TNF-α treatment increased COX-2 transcription 2-3 fold but this failed to reach statistical significance.

VCAM mRNA was significantly upregulated by LPS, IL-1β and TNF-α, compared to both saline and IL-6 challenges (p<0.001). There were not significant differences between the level of expression induced by LPS, IL-1β and TNF-α. These differences were demonstrated by Bonferroni post-hoc tests following a significant one-way ANOVA (F=11.52, df 4,19, p<0.0001). Thus, LPS, TNF-α and IL-1β administration but not IL-6 administration induced upregulation of transcription of the endothelial marker VCAM.

uPAR mRNA was robustly increased by LPS treatment in comparison to saline, IL-1β, TNF-α and IL-6 treatments (p<0.001). However, peripheral IL-1β administration also significantly increased uPAR mRNA in relation to saline treatment (p<0.05). These differences were demonstrated by Bonferroni post-hoc tests following a significant one-way ANOVA (F=27.12, df 4,19, p<0.0001).

CD11b mRNA was not significantly altered by systemic challenge (F=0.1240, df 4,17, p=0.9719). Thus, peripheral challenge with the acute pro-inflammatory challengers did not result in an increased transcription of this marker of microglial activation.

### Time-course of pro-inflammatory cytokine expression in the brain

**Figure 7:**
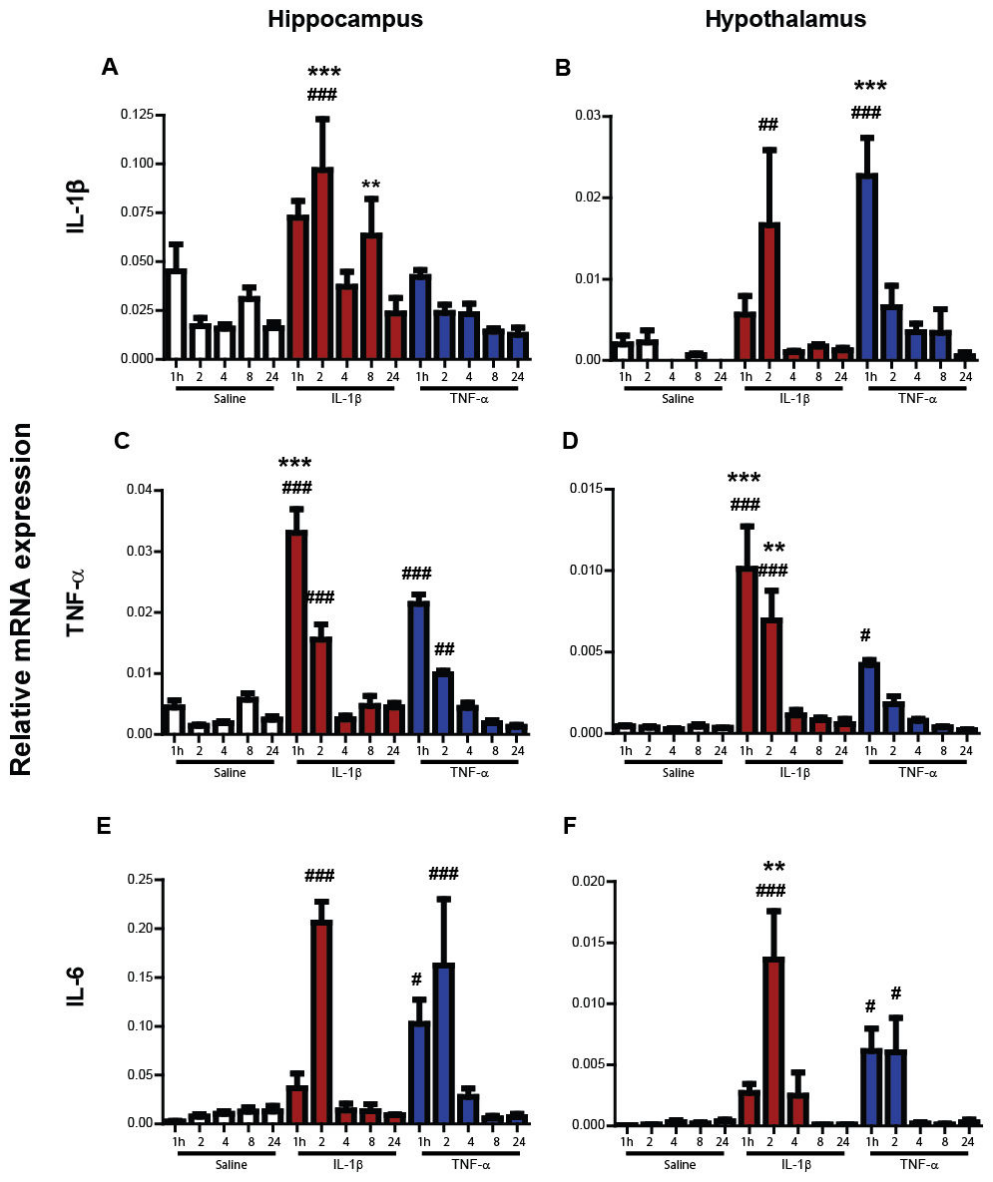
Impact of systemic IL-1β or TNF-α challenge on hippocampal and hypothalamic pro-inflammatory cytokine mRNA transcription at 1, 2, 4, 8 and 24 hours. Hippocampal and hypothalamic transcription of mRNA species for IL-1β, TNF-α and IL-6 was assessed using quantitative PCR after systemic challenge (i.p.) with saline, IL-1β (15 µg/kg) or TNF-α (50 µg/kg) at 1, 2, 4, 8 and 24 hours post-injection. All data groups were compared by two-way ANOVA, followed by Bonferroni post-hoc tests comparing differences between each IL-1β group and the equivalent saline groups and comparing the cytokines directly. # denotes IL-1β/TNF-α treatment is significantly different to equivalent saline treatment at the time point indicated; * indicates significant difference between IL-1β and TNF-α. ^#^/*p<0.05 # #/**p<0.01, ^# # #/***^p<0.001. All data have been presented as mean±SEM, n=5 for all IL-1β and TNF-α treated groups except 2 h (n=4) and saline groups n≥3.

Hippocampal and hypothalamic transcripts were assessed in IL-1β and TNF-α-treated animals at 1,2,4,8 and 24 hours (Figure 7) and compared to saline-treated animals using a two-way ANOVA analysis, with treatment as between subjects factor and time as within subjects factor, followed by Bonferroni pairwise comparisons between cytokine and saline. These revealed a significant effect of time (F≥6.64, df 4,56, p≤0.001) and treatment (F≥6.89, df 2, 56, p≤0.01) for each transcript.

Hippocampal and hypothalamic IL-1β mRNA was significantly increased in animals 2 hours after i.p. IL-1β in comparison with saline challenged animals (p<0.001 and <0.01, respectively). Transcription increased in IL-1β–challenged animals non-significantly at 1 hour when compared to saline. Elevation was relatively short lasting and levels had dropped back to basal expression at 4 hours. Interestingly, no difference could be observed in the hippocampus after peripheral TNF-α administration, but a sharp and short-lived induction of IL-1β was measured in the hypothalamus (p<0.001). Hypothalamic level of IL-1β one hour post-challenge was significantly higher after TNF-α than IL-1β (p<0.001), pointing to a different time frame of peak activity.

TNF-α mRNA was also significantly up-regulated by systemic IL-1β and TNF-α challenge compared to saline at the 1- and 2-hour time points in the hippocampus and the hypothalamus (TNF-α-induced TNF-α expression was not significant in the hypothalamus after 2 hours). TNF-α expression was higher in IL-1β challenge compared to TNF-α on the initial hour (p<0.001) in the hippocampus, and after 1 (p<0.001) and 2 (p<0.01) hours in the hypothalamus.

Hippocampal as well as hypothalamic IL-6 mRNA was induced acutely by IL-1β challenge. at 2 hours post-injection (p<0.001). TNF-α increased IL-6 expression in both regions for the first two hours following its administration (p<0.05). Peak expression of hypothalamic IL-6 in IL-1β treated animals was significantly higher than in TNF-α treated animals (p<0.01)

### Timecourse of hippocampal expression of chemokine and other inflammatory transcripts

**Figure 8:**
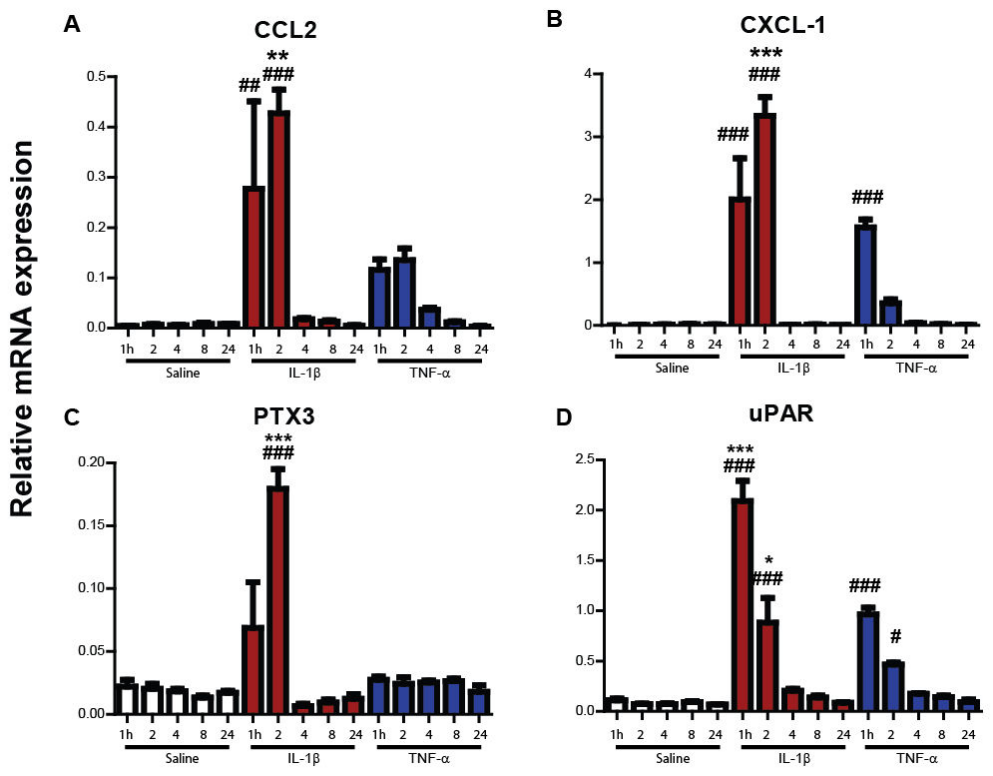
Impact of systemic IL-1β and TNF-α challenge on hippocampal cell activation and pathway markers at 1, 2, 4, 8 and 24 hours. Hippocampal mRNA transcription of CCL2, CXCL1, PTX3 and uPAR was measured using quantitative PCR after systemic challenge (i.p.) with saline, IL-1β (15 µg/kg) or TNF-α at 1, 2, 4, 8 and 24 hours post-injection. All data groups were compared by two-way ANOVA, followed by Bonferroni post-hoc tests comparing differences between each IL-1β or TNF-α group and the equivalent saline groups and comparing the cytokines directly. # denotes treatment is significantly different to saline, while * denotes a difference between IL-1β and TNF-α. #/* p < 0.05, # #/** p < 0.01, # # #/*** p<0.001. All data have been presented as mean±SEM, n=5 for all IL-1β/TNF-α groups, except all 2 hour groups (n=4). All saline groups n≥4 except 4 and 24 hours n≥3.

We assessed the temporal expression of further inflammatory transcripts in the hippocampus (Figure 8) and analysed these by two-way ANOVA as described above. CCL2 mRNA was induced both by systemic IL-1β and TNF-α but this expression was significantly greater in IL-1β-treated animals (p<0.05). Hippocampal CXCL1 mRNA was upregulated as soon as 1 hour by both systemic IL-1β and TNF-α challenges and these increases were statistically significant compared to saline at 2-hours post-challenge (p<0.001). CXCL1 expression was significantly higher after IL-1β treatment with respect to TNF-α at 2 hours (p<0.001).

PTX3 mRNA was significantly upregulated 2 hours after IL-1β challenge (p<0.001), while TNF-α did not induce any increase in this transcript.

Finally, uPAR mRNA was significantly upregulated by systemic IL-1β and TNF-α challenge compared to saline at the 1 and 2 hour time points (p<0.05). The magnitude of this effect was greater in the IL-1β group (p<0.05).

### Immunohistochemistry

**Figure 9:**
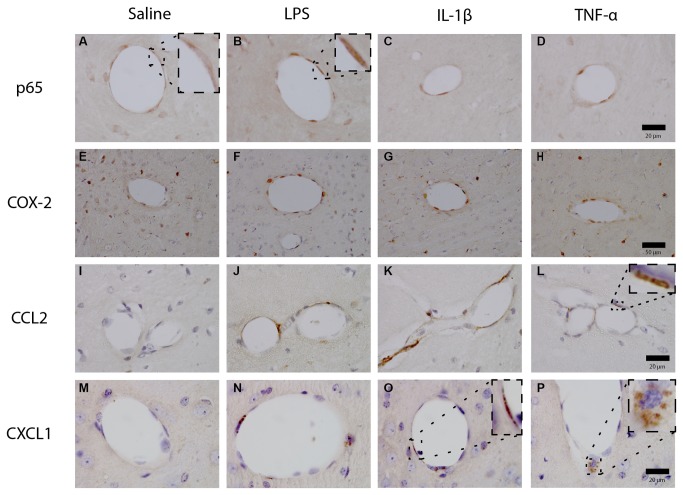
Expression of pro-inflammatory mediators at the brain vasculature following peripheral immune challenge. Representative micrographs of vascular NFκB p65, COX-2, CCL2 and CXCL1 2h after i.p. challenge with either saline, LPS (100µg/kg), IL-1β (15µg/kg) or TNF-α (50µg/kg). All pictures were taken from the hippocampus, except for COX-2, which were taken from the thalamus since neuropil labelling with COX-2 is also high in the hippocampus, thus reducing the contrast with activated endothelium. Scale bars are 20µm and 50µm as shown.

To confirm translation of some of the transcripts described above, and to identify likely sites of expression, we performed immunohistochemistry for the expression of the p65 subunit of NF-κB and for COX-2, CCL2, CXCL1, IL-1β and TNF-α in various regions of mice brain harvested two hours after a peripheral challenge with LPS (100µg/kg), IL-1β (15µg/kg), TNF-α (50µg/kg) or physiological saline (Figure 9). At this time point, we could only detect differences at or near blood vessels. These changes were generally apparent in a relatively homogenous fashion in the hippocampus, the thalamus, the hypothalamus and other regions. We have chosen representative examples from the thalamus and hippocampus.

Although we could observe positive staining for NFκB p65 in saline treated animals, it was mostly cytosolic with sparing of the nucleus (Figure 9a and inset), while inflammatory challenge was associated with a strong nuclear localisation at the cerebral vasculature (Figure 9b–d). Consistent with this, clear positive staining for COX2 was also present at the cerebrovascular endothelium in animals challenged with LPS or IL-1β (Figure 9f,g), and to a lesser extent, TNF-α (Figure 9h). That is to say, COX-2 activation was obvious at the endothelium of the vast majority of larger vessels in the LPS and IL-1β treated animals and was less strongly stained in TNF-α treated animals, but once again at most vessels. Conversely saline-treated animals did not show vascular COX-2, except for the presence of occasional COX-2 positive-perivascular macrophages, which were present in all animals. Both CCL2 and CXCL1 could rarely be detected in saline-treated mice, but LPS, IL-1β and TNF-α all induced increases in labelled cells at the cerebrovasculature. These effects were most obvious for LPS and IL-1β. Positive labelling for CXCL1 and CCL2 was significantly less frequent than that for COX-2, i.e. less positive vessels, but robust in its intensity at those vessels. CCL2/CXCL1-positive vessels also tended to have less positively labelled cells per vessel when compared to COX-2.

Chemokine expression appeared endothelial with thin elongated areas of labelling along the perimeter of the vessels of the hippocampus, thalamus and hypothalamus. However, TNF-α-induced CXCL1 expression appeared to be limited to a subpopulation of perivascular cells that was less extensively present in LPS and IL-1β stimulated animals. TNF-α, at the doses used here, appeared to be a weaker inducer of p65, COX-2, CCL2 and CXCL1 than IL-1β or LPS, although its ability to induce each of these molecules was clearly different to saline.

Expression of IL-1β and TNF-α could be detected in occasional cells of the choroidal epithelium but could not be reliably detected at the vasculature or in the brain parenchyma, despite clear labelling on positive-control slides from prion-disease (ME7) animals treated intracerebrally with TNF-α (data not shown).

**Figure 10:**
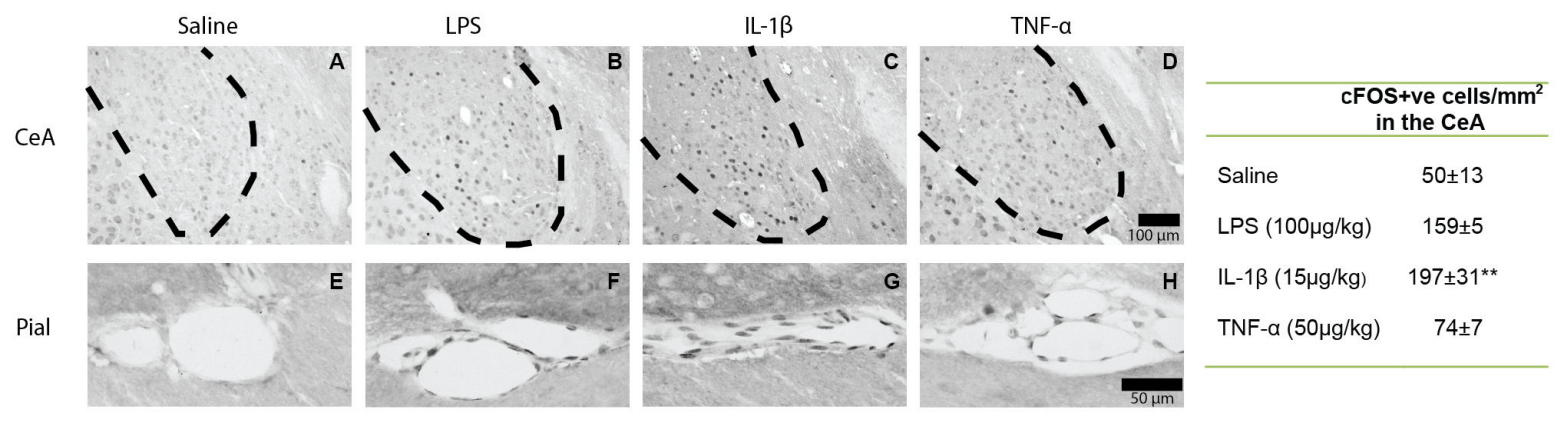
c-Fos expression in the central nucleus of the amygdala (CeA; A-D) and in the pial membrane (E–H) in brains of mice 2h after i.p. treatment with saline, LPS (100µg/kg), IL-1β (15µg/kg) or TNF-α (50µg/kg). cFOS-positive neurons in the CeA are quantified and shown in the integrated table. ** denotes statistically significant difference from saline by Bonferroni post-hoc test after significant one way ANOVA.

Systemic IL-1β and, to a lesser extent, TNF-α, induced an increase in c-FOS immunoreactivity in the central nucleus of the amygdala (Figure 10 A–D). Systemic LPS was used as positive control. The number of cFOS-positive cells was assessed by blindly counting positive cells from at least 3 10µm sections, per animal, containing the central nucleus of the amygdala (Figure 10, table). One way ANOVA analysis of cFOS counts showed that IL-1β was significantly elevated with respect to both TNF-α (p<0.05) and saline (p<0.01). TNF-α, was greater than, but not significantly different to saline (p>0.05), perhaps due to higher cFOS counts in 2 of 5 saline-treated animals. In addition to neuronal cFOS labelling there was also clear labelling of vascular associated cells present in the pial membranes, between the hippocampus and the thalamus (Figure 10 E–H), consistent with the idea that cFOS labelling is not limited to detection of early gene expression in neurons.

## Discussion

The current comparison of the acute effects of LPS, IL-1β, TNF-α and IL-6, on locomotor activity, core body temperature, plasma inflammatory mediators, hepatic, hypothalamic and hippocampal inflammatory expression profiles constitutes the most comprehensive comparative analysis performed to date. We observed that IL-1β and TNF-α induce acute systemic inflammation and reduced locomotor activity. The CNS inflammatory transcription elicited by these cytokines is brief but broadly similar to that elicited by systemic LPS, although the cytokines do not induce IFN-β mRNA. IL-1β but not TNF-α induces PTX3, while hippocampal CXCL1, CCL2, IL-6 and uPAR transcripts were produced to a greater extent by IL-1β challenge. A robust endothelial activation comprising COX-2, CCL2 and CXCL1 synthesis was confirmed by immunohistochemisty. Peripheral IL-6 was found to induce a minimal CNS response. The systematic comparison, across the blood, liver, hypothalamus and hippocampus, of the effects of LPS and three pro-inflammatory cytokines fundamental to a systemic inflammatory response [33] provides a useful resource that, while necessarily limited in depth and breadth, nonetheless functions as a relatively concise overview of key differences and similarities between responses to i.p. challenges with pro-inflammatory cytokines.

### Dose-dependence of CNS inflammatory effects

As expected, LPS induced the most widespread inflammatory profile and sickness behaviour symptoms. Since the inflammatory response generated by LPS has been reviewed in detail elsewhere [3,34], the present discussion concentrates on the responses generated by the systemic pro-inflammatory cytokines. While LPS was injected at a dose that mimics relatively mild systemic inflammation [28,35], doses for the individual immune challenges were chosen after review of the sickness behaviour literature, which has produced the majority of studies on the CNS consequences of systemic cytokines. Attempts were made to encompass the range at which the cytokines have been used across sickness behaviour studies with two doses (Table 2). The two IL-1β doses used in the comparative study elicited broadly equivalent responses. Since significantly different effects were not found between the doses this would appear to indicate that IL-1β does not require high systemic concentrations to be maximally active. This is consistent with studies which show that systemic blockade of IL-1β activity using IL-1RA abrogates symptoms in autoimmune diseases despite undetectable levels of systemic IL-1β [36]. It was of note that the higher dose of IL-1β actually induced less CXCL1 in the liver and we investigated the possibility that this may be due to more robustly elevated IL-1ra in the high dose group. However, levels of IL-1ra induced by the two IL-1β doses were not significantly different. It remains possible that other anti-inflammatory molecules such as IL-10 may have an influence in this regard. It is worth pointing out that the IL-1β plasma levels 2 hours after IL-1β injection at 50 µg/kg were approximately 10-fold higher than those induced by LPS and it was one of our aims to define doses of cytokines that were relevant to LPS-induced levels. Thus, the lower IL-1β dose (15 µg/kg) was used in assessing IL-1β effects across a 24-hour time course. The dose range for TNF-α was larger, and reflected the higher doses of TNF-α used in the literature. Some differences in effects emerged, in the induction of TNF-α mRNA in the liver, hypothalamus and hippocampus. The TNF-α inducible protein, TNF-αIP2, was induced to almost equal levels in the liver by both doses, suggesting that the excess TNF-α produced by the higher dose was not necessarily more biologically active. Thus, on most measures, the low dose of TNF-α was comparable to the high dose. Nonetheless, plasma levels of TNF-α after 250 µg/kg TNF-α, were as much as 25-fold higher than those present in the 50µg/kg-injected animals (table 3) suggesting some clearly differential effects of the higher dose. While no obvious differences were present at 2 hours, it would be of interest to know the longer-term consequences of these higher levels. Nonetheless, the levels induced by 15µg/kg TNF-α were very similar to LPS-induced levels, which was what we wished to simulate, and therefore this dose was used for subsequent time course and immunohistochemical experiments.

Given that IL-6 had no effect on most measures, there was some concern that the doses used for this cytokine were simply below those required for biological activity. However, we clearly demonstrated an induction of the liver acute phase gene SAA2, consistent with the known role of IL-6 in the hepatic acute phase response [37,38], as well as a mild effect on locomotor activity at 125 µg/kg, and thus have shown biological activity of IL-6, but very limited effects on the CNS. A comparison of the relationship between the doses of cytokines injected individually in the present study in relation to levels generated by LPS challenge (table 3) is of interest. Plasma levels of IL-1β, TNF-α and IL-6 elicited by LPS injection, seen here and in recent studies [28], indicate that TNF-α circulates at approximately a two-fold higher level than IL-1β. Here we have injected TNF-α at concentrations that were 3.5-5 times higher than those of injected IL-1β. However, IL-6 circulated at much higher levels after LPS injection (at levels 10 fold greater than TNF-α) and this argues that a higher IL-6 dose might have been required in order to replicate LPS-induced levels. Peripheral IL-6 does contribute to facets of LPS- and IL-1β-induced sickness behaviour [39] and appears to be crucial in endotoxin-mediated fever [40]. Evidence suggests that it is involved in the transport of PGE2 to its cognate receptors to initiate the fever response [41]. Thus, while elevation of IL-6 alone may not be sufficient to elicit neuroinflammation, it does appear to synergise with other cytokines. Unlike IL-1β and TNF-α, peripheral IL-6 challenge fails to elicit endothelial cell NFκB signalling or COX-2 transcription [42,43] and it is possible that the induction of IL-6R and gp130 by other inflammatory mediators, such as LPS, TNF-α or IL-1β, is necessary for IL-6 signalling at the brain microvasculature [44].

### IL-1β and TNF-α elicit systemic and central inflammatory changes

Peripheral injection of IL-1β and TNF-α induced a number of measures of systemic inflammation. IL-1β induced no significant circulating TNF-α, whereas TNF-α did induce circulating IL-1β protein. This was demonstrated both in the comparative study and the IL-1β time course study. The cytokine signalling system is complex and context dependent [45], but TNF-α is known to precede induction of IL-1β in lethal bacteraemia [46]. In that study the authors, using a TNF antibody, show that TNF-α is necessary for the subsequent release of IL-1β. Fantuzzi and Dinarello have shown that IL-1β-deficient mice produce the equivalent levels of TNF-α to wild type animals following LPS challenge [47]. Moreover, IL-1RA administration with LPS does not alter circulating TNF-α [48]. Collectively these data indicate that TNF-α does indeed induce circulating IL-1β but that IL-1β does not induce circulating TNF-α. It is important to stress that this relationship differs when it comes to CNS induction of cytokine transcripts by these cytokines: TNF-α can induce CNS expression of IL-1β transcription in both the hypothalamus and the hippocampus, while systemic IL-1β robustly also induced TNF-α mRNA in both areas. Both systemic cytokines can also induce their own transcription in the CNS.

It is of interest that LPS, but not any of the cytokines, induced an increase in circulating PGEM, measured as a proxy for the rapidly metabolized PGE2, while LPS, IL-1β and TNF-α appeared approximately equally capable of inducing corticosterone. Several aspects of the sickness and stress responses have implicated peripherally generated prostaglandins [49] and we have previously shown that this rapid systemic PGE2 response is COX-1-mediated [50]. It will be of interest to examine the role of systemic prostaglandins in LPS-induced cognitive deficits [26,28].

While both pro-inflammatory cytokines induced pro-inflammatory gene transcription in the hippocampus, IL-1β did appear to be more potent than TNF-α in induction of IL-6, CCL2, and CXCL1. Indeed CXCL1 induction following IL-1β was also significantly greater than that seen after LPS challenge, suggesting that it is induced by IL-1β and that levels resulting from LPS may be dependent on first inducing IL-1β. However, experiments with systemically administered IL-1ra will be necessary to confirm or refute this. The current data indicate an activation of the brain endothelium, with hippocampal induction of VCAM, uPAR and COX-2 mRNA and the more robust activation by IL-1β appears to be borne out also by immunohistochemisty. Here we have demonstrated nuclear translocation of NFκB p65 subunit at the brain endothelium, as has previously been demonstrated after IL-1β challenge [51] and consequent COX-2, CCL2 and CXCL1 labelling, all in the cerebrovasculature. Receptors for both TNF-α and IL-1β have been demonstrated on endothelial cells [43] and both these and perivascular macrophages are highly receptive to circulating IL-1β and TNF-α [52], transducing inflammation into the brain parenchyma via NFκB activation [53], PGE2 synthesis [54] and contributing to sickness behaviour [55]. While the observed upregulation of uPAR transcription offers evidence of microglial activation [32], other markers of microglial activation, CD11b and COX-1 were not elevated at 2 hours. It is clear that uPAR can also be expressed by endothelial cells [56,57] and is induced by LPS [58]. The robust induction by IL-1β compared to that by TNF-α at 2 hours was striking but full time course examination revealed that both IL-1β and TNF-α can induce uPAR in the CNS, though IL-1β-induced uPAR remained more robust and this is a novel finding. Collectively, the changes in expression of uPAR, VCAM and COX-2, with little or no change in COX-1 and CD11b are likely to be descriptive of a predominantly endothelial response to these systemic cytokine challenges. The robust expression of c-FOS in the pial membranes also suggests activation via the ventricular system. These conclusions are supported by the COX-2 and chemokine immunohistochemistry presented here. It is important to note that systemic LPS can signal to the brain endothelium without the need for systemic cytokine synthesis [59]. Thus irrespective of other routes of communication, circulating PAMPs and/or mediators are sufficient to transduce systemic inflammatory signals to the brain. The route of administration used in the current study is likely to contribute to the brain centres activated. It is clear that intraperitoneal injection will lead to activation of vagal afferents [60] and this route could potentially have been by-passed by intravenous challenge. Activation of the central nucleus of the amygdala, shown here by nuclear cFOS labeling after LPS, IL-1β and TNF-α is likely to reflect a neural route from the periphery to the CNS, since vagotomy clearly decreases LPS/IL-1-induced cFOS in the amygdala [60,61]. Given the clear endothelial response observed here, and the measurable systemic cytokine levels, it is likely that cytokines, both those injected and those synthesized locally, have leaked out of the peritoneal cavity and been systemically disseminated. Therefore, both neural and humoral routes are occurring in the current studies, but the endothelial activation is occurring despite the route of inoculation rather than because of it, while the neural route is a certainty after intraperitoneal challenge. The amygdaloid neurons activated in the current study do not express TLR4 [62] and are thus not directly responsive to LPS, but both LPS- and IL-1β-induced cFOS activation and locomotor hypoactivity are reduced by vagotomy [61]. It is known that activation of the amygdala impacts on frontocortical function and increased anxiety and loss of frontocortical influence may be important aspects of depression and delirium [63].

Returning to evidence for the activation of the cerebral endothelium, only LPS challenge elicited IFN-β induction, and there was neither central nor peripheral upregulation of type-I IFNs after IL-1β or TNF-α challenge. These results support previous observations that neither cytokine elicits IRF3 phosphorylation or IRF3-dependent gene transcription [64,65], which have a central role in IFN-β gene induction [66] and those by our own laboratory [28] suggesting that induction of central IFN-β transcription by systemic LPS results from the direct stimulation of the endothelial cell TLR4 MyD88-independent pathway [67]. The present results also demonstrate that systemic IL-1β and TNF-α cannot elicit a peripheral or central type-I IFN response.

IL-1β but not TNF-α challenge induced hippocampal PTX3. We have previously suggested that PTX3 is induced downstream of IL-1β and is thus a useful indicator of IL-1β protein synthesis and action [30] and the current data would suggest that, at least in the CNS, this is correct. It is of interest that PTX3 has recently been associated with dementia progression [68] and whether the latter is linked to its synthesis downstream of IL-1β merits investigation. Notably, a recent *in vitro* study has demonstrated that elevated PTX3 impedes microglial phagocytosis of apoptotic neurons [69] and our own data have indicated that IL-1β synthesis and phagocytic activity in microglia in the degenerating brain represent divergent pathways of activation [70].

### CNS expression of chemokines

The current data show a clear acute CNS induction of the chemokine transcripts CXCL1 and CCL2 after LPS, IL-1β and TNF-α, and both CCL2 and CXCL1 protein were clearly induced in the brain vasculature following peripheral challenge with LPS, IL-1β and TNF-α, although less robustly by TNF-α. The up-regulation of these chemokines is notable. Firstly they are key in the recruitment of immune cells to the brain: CCL2 mainly attracts monocytes cells (macrophages and microglia), and CCL2 injection into the hippomcapus induces monocytic cells accumulation with greater potency than other monocyte chemokines [71]. CXCL1 is chemoattractant towards neutrophils and is the rodent homolog of human IL-8 [72]. There are also indications that some chemokines, and especially CCL2, contribute to weakening the blood brain barrier, allowing macromolecules that are normally sequestered, such as cytokines, and cells such as leukocytes to more freely cross into the brain [73,74]. The action of CCL2 on blood brain barrier disruption appears to be directly mediated by CCR2 expression in endothelial cells [75]. Finally, chemokines have recently been recognized as being able to directly modulate neuronal activity [76,77]. Accordingly, CCL2 and CXCL1, as well as their respective receptors CCR2 and CXCR2, are all present in neurons and glial cells [78–80]. CCL2 has been shown to activate p38 MAPK, as well as its downstream transcription factor CREB, in hippocampal cultures [81]. Moreover, short term potentiation was altered in hippocampal slices from transgenic mice over-expressing CCL2 in astrocytes [82], and CCL2 enhances neuronal excitability and synaptic transmission in rat hippocampus [83]. The role of CXCL1 in brain function has, so far, generated less interest, but peripheral administration of CXCL1 results in a reduction in open field activity and burrowing [84] and its acute elevation in plasma in the IL-1β time course is consistent with the cytokine being a plausible mediator of CNS activation. It has been shown to enhance neurotransmitter release and impair the long term depression of synaptic strength of Purkinje neurons [85]. Similarly, CXCL-1 stimulates MAPK and PI3K-mediated growth factor signalling in cortical neurons [86]. There is also evidence of direct effects of IL-8 on septal cholinergic neurons [87]. Although there is evidence to suggest that central CXCL1 may be neuroprotective [88] it has been shown that activation of its receptor CXCR2 is neurotoxic at high chemokine concentrations [89].

### Implications for clinical CNS conditions

With the emerging literature on impacts of systemic cytokines on CNS disorders, it is of interest to discuss the current data in the context of that literature, although some limitations have to be acknowledged. The current experiments were performed with all female mice and it is observed that gender affects susceptibility to endotoxemia. Male sepsis patients have a mortality rate nearly 3 times higher than age- and disease severity-matched female patients [90]. A similar ratio was reported in rodents receiving intravenous injection of LPS [91] associated with an higher production of TNF-α [92], IL-1β [93] and IL-6 [94] after LPS challenge in male mice. We would thus anticipate a higher response if we had used male mice. Not only gender, but timing in the oestrus cycle, can also modulate the murine response to an immune challenge, with diestrus being associated with a stronger response than proestrus [95]. It is possible that some of the differences observed in the current study could be accounted for by the fact that our mice were not synchronized with respect to the oestrus cycle. However, the patterns of expression observed here are largely binary (ie robustly expressed or not expressed) and therefore unlilkely to have been fundamentally altered by oestrus status. It might be predicted that failure to synchronise for oestrus might have particularly affected the plasma corticosterone levels, which were not significantly increased by treatments in the current study and which are known to be highest during proestrus and lowest during diestrus [96,97].

There are also significant differences between humans and rodents with respect to immune responses to both infectious and sterile inflammatory insults. It has recently been reported in microarray studies that the systemic inflammatory response syndrome to burns, trauma and endotoxemia in humans is highly stereotyped but is dramatically different to the immune response to those insults in mouse, with a correlation of less than 0.01 across 5,544 genes showing 2-fold altered expression [98]. While this might suggest that the immune responses are fundamentally different between these species, the correlations improve when observing the genes showing the greatest elevation. Furthermore, studies comparing the primary response genes analysed in the current experiments show much more similar responses to endotoxin between human and mouse [99].

Finally, it has to be stated that the cytokine challenges made in the current study were acute and levels would have returned to basal levels within hours. This maybe particularly true for IL-6 which was significantly below LPS-induced levels after just 2 hours. Nonetheless, when put in the context of human studies which measure elevated peripheral IL-6 in depression, cognitive decline and CNS disease [10,11,100–102] the current results are not consistent with direct CNS inflammatory effects of systemic IL-6. Since IL-6 is typically produced at high levels in response to acute inflammation, it may be that these higher levels simply make it the inflammatory cytokine most likely to be measurable in a number of settings. As such its presence may be indicative of increased severity of systemic inflammation in a general sense rather than IL-6 itself being the key mediator of the CNS effects of this more severe inflammation. It is now known that plasma IL-1β is not detectable in many auto-inflammatory diseases that are nonetheless mediated by IL-1β and treatable with IL-1ra [103]. Given the results of the present study, it seems more likely that the CNS inflammatory response to acute inflammatory events elicited by infection, surgery or trauma, is being driven by TNF-α or IL-1β, rather than IL-6. Nonetheless, with the increasing evidence that moderate systemic inflammation has negative consequences for patients with ongoing neurodegeneration [27] systemic IL-6 may signal different responses in those with prior susceptibilities or may synergize with other inflammatory mediators to actually contribute to these disease exacerbations. Furthermore, chronically elevated IL-6 may represent an entirely different scenario. en if IL-6 itself does not drive CNS inflammation, it remains useful as a measurable biomarker of potentially deleterious acute systemic inflammation in populations with ongoing CNS pathology.

Cognitive consequences of systemic inflammation have emerged in recent years [104,105] and most of these studies have implicated hippocampal expression of pro-inflammatory cytokines as a key step in inducing neuronal dysfunction. Both IL-1 and TNF-α can directly impact neuronal and synaptic functioning in the hippocampus [106,107] and both cytokines have also been shown to negatively affect memory function [108–110]. In a model of peripheral surgery-induced cognitive impairment, the memory dysfunction elicited following tibial fracture was shown to be both TNF-α and IL-1β-dependent [111,112]. . Moreover, the resultant central IL-1β elevation was shown to be downstream of peripheral TNF-α [112]. Deficits in the contextual fear conditioning paradigm have been clearly shown to rely on direct action of IL-1β on neurons of the hippocampus but it remains unclear how cognitive impairment occurs after systemic inflammation in most paradigms. Other studies with systemic inflammation, induced by LPS, have implicated IL-6 in the cognitive effects [113] and raised systemic IL-6 has been associated with post-hip fracture delirium [22]. The current studies would suggest that this systemic IL-6 is unlikely to produce cognitive dysfunction via the induction of CNS inflammatory pathways and it is also clear that while elevated IL-6 was associated with delirium, high IL-6 was present in many patients who did not become delirious, and indeed that prior cognitive impairment was the stronger predictor of delirium. This raises an important issues for all studies of CNS disorders associated with systemic inflammatory mediators: systemic inflammation has significantly different effects on the brain contingent on the vulnerability of the brain at the time of the systemic inflammatory event. Systemic inflammation induced by LPS is particularly damaging when it interacts with chronic neurodegeneration, producing exaggerated hippocampal inflammation [114], acute cognitive impairment [28] and acceleration of disease [35]. Ageing also confers increased vulnerability on the hippocampus to the consequences of systemic inflammatory challenges [115–118]. Low-level systemic inflammation, produced by infection, surgery or tissue trauma, has been shown to induce exaggerated sickness behaviour symptoms [119], precipitate episodes of delirium [120] and contribute to progression of cognitive decline [19] in aged and demented patients. Central to many of these observations may be the finding that microglia in the aged and diseased brain are primed to produce exaggerated inflammatory responses to systemic inflammatory insults, producing significantly elevated CNS IL-1β [114]. In the current study the neuroinflammatory profile after peripheral IL-1β and TNF-α challenges returned to baseline within 4 hours of challenge but the challenges used in the current study were predicted to produce mild *adaptive* responses in the CNS. We have hypothesized that similar challenges produce a *maladaptive* sickness response in the aged or demented [63]. Thus systemic inflammation, which is not sufficiently severe to induce CNS dysfunction in most individuals, clearly is sufficient in those with prior cognitive vulnerability and this represents an important route to understanding the significance of systemically elevated inflammatory mediators for CNS disorders.
