# Correction: A Systematic Analysis of the Peripheral and CNS Effects of Systemic LPS, IL-1β, TNF-α and IL-6 Challenges in C57BL/6 Mice

**DOI:** 10.1371/annotation/90c76048-2edd-4315-8404-4d9d8cbd411e

**Published:** 2013-12-09

**Authors:** Donal T. Skelly, Edel Hennessy, Marc-Andre Dansereau, Colm Cunningham

An error was introduced into the title of the article during preparation for publication. The greek symbol beta in IL-1β was incorrectly substituted with the letter B. The correct title is: A Systematic Analysis of the Peripheral and CNS Effects of Systemic LPS, IL-1β, TNF-α and IL-6 Challenges in C57BL/6 Mice. The correct citation is: Skelly DT, Hennessy E, Dansereau M-A, Cunningham C (2013) A Systematic Analysis of the Peripheral and CNS Effects of Systemic LPS, IL-1β, TNF-α and IL-6 Challenges in C57BL/6 Mice. PLoS ONE 8(7): e69123. doi:10.1371/journal.pone.0069123

